# Nuclear-encoded mitochondrial MTO1 and MRPL41 are regulated in an opposite epigenetic mode based on estrogen receptor status in breast cancer

**DOI:** 10.1186/1471-2407-13-502

**Published:** 2013-10-27

**Authors:** Tae Woo Kim, Byungtak Kim, Ju Hee Kim, Seongeun Kang, Sung-Bin Park, Gookjoo Jeong, Han-Sung Kang, Sun Jung Kim

**Affiliations:** 1 Department of Life Science, Dongguk University-Seoul, Seoul 100-715, Korea; 2 Research Institute and Hospital, National Cancer Center, Gyeonggi do 411-764, Korea

**Keywords:** Breast cancer, Epigenetics, Estrogen receptor, MRPL41, MTO1

## Abstract

**Background:**

MTO1 and MRPL41 are nuclear-encoded mitochondrial genes encoding a mitochondrial tRNA-modifying enzyme and a mitochondrial ribosomal protein, respectively. Although both genes have been known to have potential roles in cancer, little is known about their molecular regulatory mechanism, particularly from an epigenetic approach. In this study, we aimed to address their epigenetic regulation through the estrogen receptor (ER) in breast cancer.

**Methods:**

Digital differential display (DDD) was conducted to identify mammary gland-specific gene candidates including MTO1 and MRPL41. Promoter CpG methylation and expression in breast cancer cell lines and tissues were examined by methylation-specific PCR and real time RT-PCR. Effect of estradiol (E2), tamoxifen, and trichostatin A (TSA) on gene expression was examined in ER + and ER- breast cancer cell lines. Chromatin immunoprecipitation and luciferase reporter assay were performed to identify binding and influencing of the ER to the promoters.

**Results:**

Examination of both cancer tissues and cell lines revealed that the two genes showed an opposite expression pattern according to ER status; higher expression of MTO1 and MRPL41 in ER- and ER+ cancer types, respectively, and their expression levels were inversely correlated with promoter methylation. Tamoxifen, E2, and TSA upregulated MTO1 expression only in ER+ cells with no significant changes in ER- cells. However, these chemicals upregulated MRPL41 expression only in ER- cells without significant changes in ER+ cells, except for tamoxifen that induced downregulation. Chromatin immunoprecipitation and luciferase reporter assay identified binding and influencing of the ER to the promoters and the binding profiles were differentially regulated in ER+ and ER- cells.

**Conclusions:**

These results indicate that different epigenetic status including promoter methylation and different responses through the ER are involved in the differential expression of MTO1 and MRPL41 in breast cancer.

## Background

The estrogen receptor (ER) plays key roles in breast cancer development and progression [1,2]. Thus, key areas of study in breast cancer are those mechanisms that regulate ER expression in normal and malignant breast tissues. Recent studies have shown that gene expression profiles differ according to hormone receptor status of the breast cancer [3,4]. ER status also affects the DNA methylation state of a wide range of genes such as FAM124B, ST6GALNAC1, NAV1, and PER1 in breast cancer [5]. These genetic and epigenetic alterations in ER + tumors make them more sensitive to endocrine therapy, whereas ER- tumors are hormone independent [6,7].

MTO1 and MRPL41 are nuclear-encoded mitochondrial genes located at 6q13 and 9p34, respectively. MTO1 encodes an enzyme involved in post-transcriptional modification of mitochondrial tRNAs (mt-tRNAs) [8]. In both humans and yeasts, MTO1 increases the accuracy and efficiency of mtDNA translation by catalyzing the 5-carboxymethylaminomethylation of the wobble uridine base in three mitochondrial tRNAs such as mt-tRNA^Gln^, mt-tRNA^Glu^, and mt-tRNA^Lys^[9]. A few potentially pathogenic variants of MTO1 have been identified in patients with mitochondrial disorders [10]. However, its expression and regulatory mechanism in breast cancer has not been determined.

MRPL41 (also known as BMRP) encodes a mitochondrial ribosomal protein that induces apoptosis in P53-dependent and independent manners via BCL2 and caspases in lymphoma [11]. Ectopic expression of MRPL41 induces cell death in several mammalian cell lines including primary embryonic fibroblasts of mice and human origin, and in NIH/3T3 cells, which is counteracted by BCL-2 [12,13]. The MRPL41 protein is localized in the mitochondria, stabilizes the p53 protein, and enhances its translocation to the mitochondria, thereby inducing apoptosis. Interestingly, MRPL41 stabilizes the p27 (Kip1) protein in the absence of p53 and arrests the cell cycle at the G1 phase. These results suggest that MRPL41 plays an important role in p53-induced mitochondrion-dependent apoptosis and that MRPL41 exerts a tumor-suppressive effect in association with p53 and p27. MRPL41 is downregulated in breast and kidney cancer cell lines and in tissues supporting its role as a tumor-suppressor [14].

Although MTO1 and MRPL41 have potential roles in human diseases, little is known about their molecular mechanism, particularly from an epigenetic approach. In this study, we examined the regulation of MTO1 and MRPL41 in ER+ and ER- breast cancer cells, and also in cells treated with estradiol (E2) and tamoxifen. We further investigated whether their regulation involved an epigenetic mechanism. Our present data show that methylation was inversely correlated with the differential expression. Moreover, the histone deacetylase inhibitor trichostatin A (TSA) increased MTO1 and MRPL41 expression in ER- and ER+ breast cancer cells, respectively. We found that ER differentially bound to the half-estrogen responsive elements at the promoter of both genes in ER+ and ER- cells.

## Methods

### In silico mining of breast cancer-specific genes

Digital differential display (DDD) was conducted (http://www.ncbi.nlm.nih.gov/UniGene/ddd.cgi) to identify mammary gland-specific gene candidates. We compared expressed sequence tag (EST) libraries from human breast tissues and those from various other somatic tissues. Of the genes that were overrepresented in breast tissue-derived libraries, ESTs of which the epigenetic regulatory mechanism has not yet been addressed were selected for further analysis.

### Study subjects

All patients provided written informed consent to donate removed tissue to the National Cancer Center (NCC) in Korea and samples were obtained according to protocols approved by the Research Ethics Board of NCC. Forty-eight pairs of breast cancers (BrCa) and their corresponding adjacent normal tissue specimens were obtained from patients who had undergone surgery between 2010 and 2011 at NCC. BrCa specimens were subjected to histological examination by an expert pathologist for independent confirmation of ER expression grade. The ER expression grades were scored by the Allred scoring system and varied between specimens, with a composite score ranging from 0 to 7. The average ER expression grade of the specimens with reported scores was 4.1. Specimens showing an ER expression grade > 3 were considered ER+. As chemo- and radiotherapy have previously been implicated in altering methylation patterns, no subjects who had received either type of treatment were included in the study.

### Cell culture and treatment of chemicals

The breast cancer cell lines MCF7 (ER+), T47D (ER+), MDA-MB-231 (ER-), and BT-549 (ER-) were purchased from the American Type Culture Collection (Manassas, VA, USA) and grown in Dulbecco’s modified Eagle’s medium supplemented with 10% fetal bovine serum. 5-Aza-2’-deoxycytidine (Sigma, St. Louis MO, USA), a methyltransferase inhibitor, was added to the culture medium at 5 μM for 72 hr to induce demethylation of the cytosine residues, and the medium was changed every 24 hr. E2 (Sigma) and tamoxifen (Sigma) were treated at final concentrations of 1 nM and 1 μM for 24 hr, respectively.

### Isolation of genomic DNA and total RNA

To isolate chromosomal DNA from breast tissue, approximately 50–100 mg of tissue was extracted using a genomic DNA purification kit (Promega, Madison, WI, USA) according to the manufacturer’s protocol. The extracted DNA was eluted with 250 μl of distilled water. Total RNA from breast tissue was prepared using Trizol according to the manufacturer’s protocols (Gibco BRL, Carlsbad, CA, USA). Genomic DNA and total RNA from cultured cells were prepared using an AllPrep DNA/RNA Mini kit (Qiagen, Valencia, CA, USA) with elution of 100 and 30 μl, respectively.

### Methylation-specific polymerase chain reaction (PCR) and bisulfite sequencing

Sodium bisulfite modification of genomic DNA was carried out using an EpiTect Bisulfite kit (Qiagen) according to the manufacturer’s protocol using 0.1 mg of purified DNA. The design of the MTO1 and MRPL41 PCR primers (Additional file 1: Table S1) and quantitative PCR were carried out as described previously [15]. Briefly, primer sequences were designed using the Methprimer program (http://www.urogene.org/methprimer/index1.html). Quantitative PCR was performed using a Power SYBR Green Kit (Applied Biosystems, Foster City, CA, USA) according to the manufacturer’s protocol. A methylation index was calculated for each sample using the following formula: methylation index = 1 / [1 + 2^−(CTu − CTme)^] × 100%, where CTu is the average cycle threshold (CT) obtained from duplicate quantitative PCR analyses using the unmethylated primer pair, and CTme is the average CT obtained using the methylated primer pair.

For sequencing of the methylated sites, the bisulfite-treated DNA was subjected to PCR to amplify the region. The primer sequences used were listed in Additional file 1: Figure S1. The PCR conditions were 94°C for 2 min, followed by 30 cycles of 94°C for 20 s, 55°C for 20 s and 72°C for 30 s, with a final extension at 72°C for 5 min. The resulting products were purified using a Qiaex II gel extraction kit (Qiagen) and then subjected to direct sequencing in both direction. The methylation ratio of each CpG site for each tissue was calculated as the percentage of methylation versus the methylated plus unmethylated sites.

### Quantitative real-time reverse transcription (RT)-PCR analysis

MTO1 and MRPL41 expression levels were measured by quantitative real-time RT-PCR analysis using cDNA synthesized from 5 μg of total RNA and a reverse transcription kit (Toyobo, Osaka, Japan). One microliter of cDNA was used for the PCR, and duplicate reactions were performed for each sample using a Kapa SYBR Fast qPCR Kit (Kapa Biosystems, Woburn, MA, USA) with gene-specific primers on an ABI 7500 instrument (Applied Biosystems). The primers used for these selected genes are listed in Additional file 1: Figure S1. RNA quantity was normalized to GAPDH content, and gene expression was quantified according to the 2^-ΔCt^ method [15].

### Chromatin immunoprecipitation-PCR (ChIP-PCR)

ChIP assays were performed using an EZ ChIP Chromatin Immunoprecipitation kit (Millipore, Billerica, MA, USA) as described in the supplier’s protocol. Briefly, the cross-linked chromatin was sonicated after cell lysis and then incubated with antibodies against ER (Millipore) at 4°C overnight. The immunocomplex was precipitated with Protein A-agarose (Millipore), and the beads were washed, sequentially treated with 10 μl of RNase A (37°C for 30 min) and 75 μl of Proteinase K (45°C for 4 h), and incubated at 65°C overnight to reverse cross-link the chromatin. The DNA was recovered by phenol-chloroform extraction and coprecipitation with glycogen, and dissolved in 50 μl of Tris-EDTA (TE) buffer. DNA associated with the ER was amplified by PCR using 1 μl of the precipitated DNA. PCR primers (sequences are in Additional file 1: Figure S1) were designed to amplify the ER-responsive elements (EREs) at the promoter. The PCR conditions were 30 cycles at 94°C for 40 s, 57°C for 1 min, and 72°C for 40 s.

### Luciferase assay

The upstream region of MTO1 and MRPL41 was amplified by PCR from human chromosomal DNA and cloned into the *Mlu*I and *Hin*dIII sites of pGL2Basic luciferase vector (Promega). The PCR was performed using primers (Additional file 1: Figure S1) with 35 cycles at 94°C for 30 seconds, 55°C for 1 minute, then 72°C for 2 minutes. 100 ng of the recombinant luciferase expression vector was transiently transfected into 1 × 10^4^ cells in 96-well culture plates using a transfection kit (Qiagen). Luciferase activity was measured 36 hours after transfection in three independent cultures using a dual-luciferase reporter assay system kit (Promega) on a Molecular Devices Filter Max F3 (Sunnyvale, CA, USA). The activity from the promoter spanning R0 ~ R4 of MTO1 and R0 ~ R6 of MRPL41 was normalized with that from the promoter containing only R0 fragment of each gene.

### Statistical analysis

Student’s *t*-test was used to detect differences in the methylation and expression level between normal and cancerous tissues and between ER+ and ER- tissues using SPSS for Windows, release 17.0 (SPSS Inc., Chicago, IL, USA). P-values < 0.05 were considered significant.

## Results

### MTO1 and MRPL41 show opposite methylation and expression in ER + and ER- breast cells

**Figure 1:**
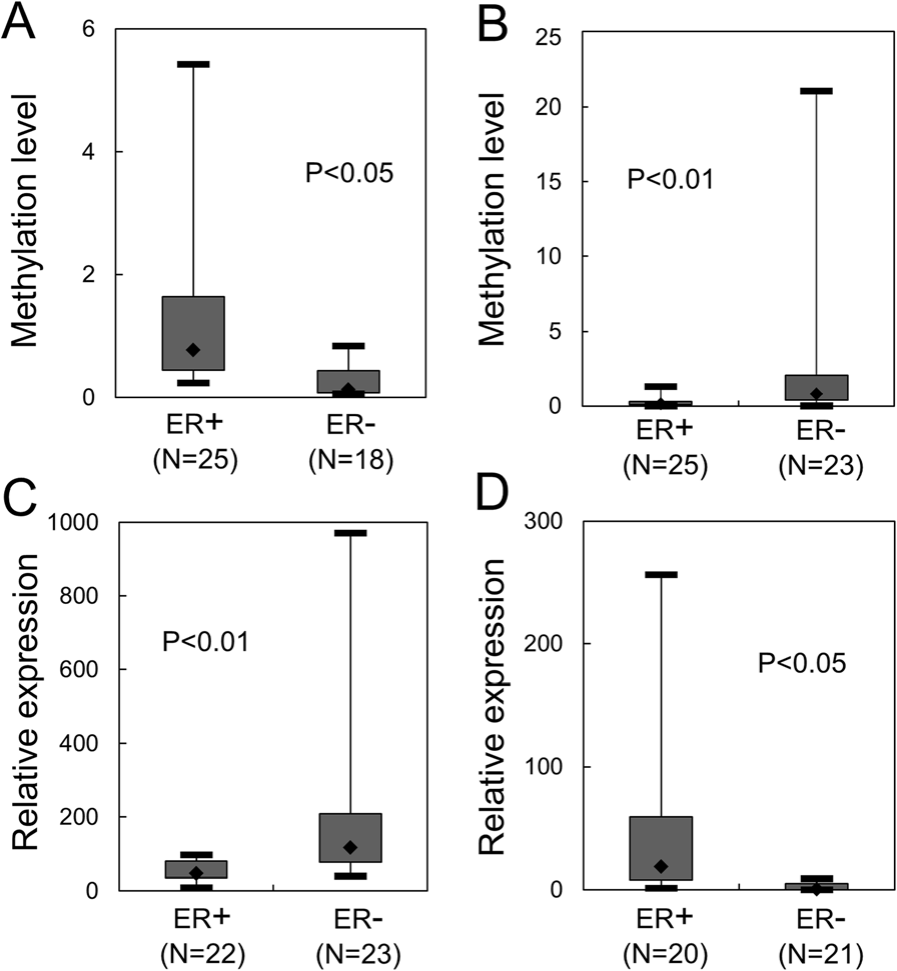
**Opposite methylation and expression patterns of MTO1 vs. MRPL41 according to estrogen receptor (ER) status in breast cancer tissues.** Methylation and expression of MTO1 **(A and C)** and MRPL41 **(B and D)** were examined by real-time methylation-specific polymerase chain reaction (PCR) and RT-PCR, respectively, in ER(+) and ER(-) breast cancer tissues. Numbers in parentheses denote the number of examined tissues. Each sample was examined in duplicate, and the average was applied to the plot. Data for individual patients is shown in Additional file 3: Figure S1.

DDD was conducted to identify genes that are abnormally expressed in breast cancer, and MTO1 and MRPL41 were identified to be expressed abundant in mammary gland with upregulation in cancer tissue (Additional file 2: Table S2). To confirm upregulation in cancer, MTO1 and MRPL41 expression was examined by real-time RT-PCR in breast cancer tissues and nearby normal tissues. However, the results revealed no statistically significant expression difference between cancer tissues and normal tissues for both MTO1 and MRPL41. Instead, expression differences emerged according to the ER status of the cancer tissues (Figure 1 and Additional file 3: Figure S1). Interestingly, the two genes showed an opposite pattern with MTO1 showing downregulation (p < 0.01) and MRPL41 showing upregulation (p < 0.05) in ER + tissues compared to ER- tissues. These results led us to explore the molecular mechanism underlying this differential expression based on ER status.

We focused on the epigenetic mechanism including DNA methylation and histone modification at the promoter. First, CpG methylation at the promoter was examined for ER+ and ER- cancer tissues by methylation-specific PCR. As shown in Figure 1, methylation level was inversely correlated with expression level; MTO1 showed higher CpG methylation but lower expression in ER + cancer tissues than in the ER- cancer tissues. MRPL41 showed lower CpG methylation but higher expression in ER + cancer tissues than in ER- cancer tissues.

**Figure 2:**
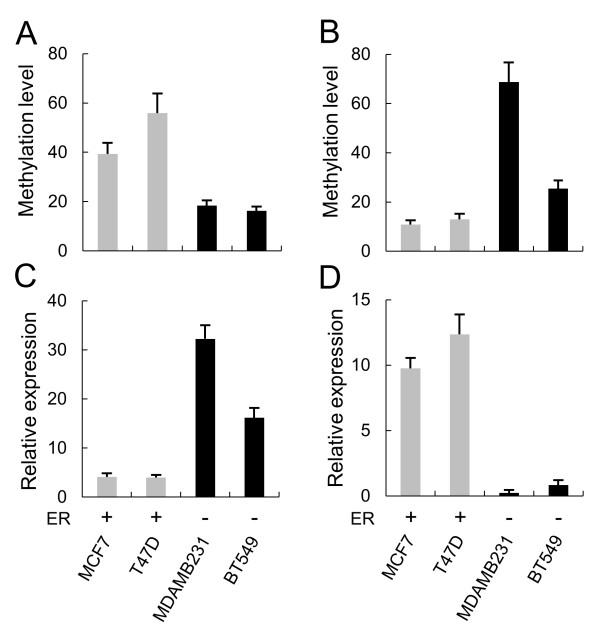
**Opposite methylation and expression patterns of MTO1 vs. MRPL41 according to estrogen receptor (ER) status in breast cancer cell lines.** Methylation and expression of MTO1 **(A and C)** and MRPL41 **(B and D)** were examined by real-time methylation-specific polymerase chain reaction (PCR) and RT-PCR, respectively, in ER(+) and ER(-) breast cancer cell lines. Each sample was examined in three independent reactions, and the average relative level is presented with the standard error.

Next, the opposite expression patterns and methylation relationships were further examined in ER+ and ER- breast cancer cell lines. The results indicated that the expression and methylation profiles in the cancer cell lines were the same as those in cancer tissues, although the overall methylation level between the cells and tissues was different (Figure 2). Further examination of the CpG sites by bisulfite sequencing confirmed the opposite methylation profile of the two genes in the ER+ and ER- cells (Additional file 4: Figure S2A and B). However, unrelated genes, A1BG and ETAA1 in the Additional file 2: Table S2, which appeared downregulated in breast cancer showed no methylation difference according to ER status as shown in the Additional file 4: Figure S2C. Therefore, MTO1 and MRPL41 were regulated by methylation in opposite manners depending on ER status.

**Figure 3:**
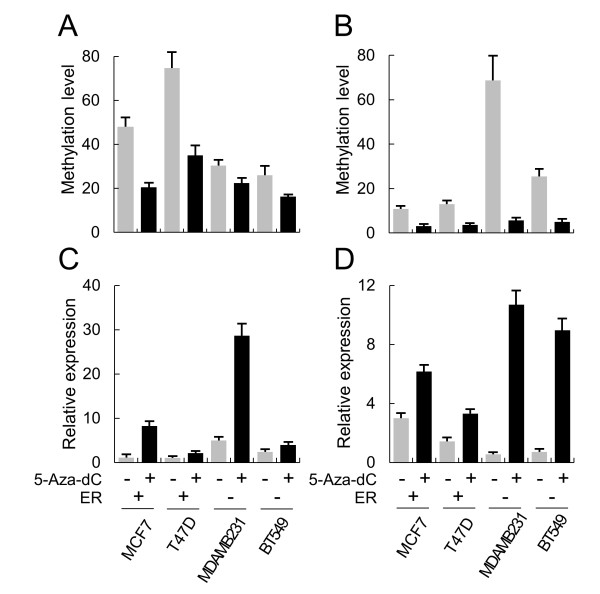
**5-Aza-dC induced upregulation of MTO1 and MRPL41.** Cultured breast cancer cells were treated with 5-Aza-dC, and methylation and expression levels were examined for MTO1 **(A and C)** and MRPL41 **(B and D)** by real-time methylation-specific polymerase chain reaction (PCR) and RT-PCR, respectively. Estrogen receptor (ER) status of each cell line is indicated as (+) or (-). Gray and black bars represent before and after treatment with 5-Aza-dC, respectively. Each sample was examined in three independent reactions, and the average relative level is presented with the standard error.

To address the effect of promoter methylation on gene expression, the methyltransferase inhibitor 5-Aza-dC was added to the cancer cell lines, and methylation and expression levels were monitored by methylation-specific PCR and RT-PCR, respectively. 5-Aza-dC induced demethylation of the two genes in cells, particularly in ER+ or ER- cells that showed higher methylation for each gene (Figure 3). RT-PCR indicated that the expression levels increased in drug-treated cells regardless of cell type. This result suggests that differential promoter methylation contributes, at least in part, to the opposite regulation of MTO1 and MRPL41.

### MTO1 and MRPL41 are oppositely regulated by E2, tamoxifen, and trichostatin A

**Figure 4:**
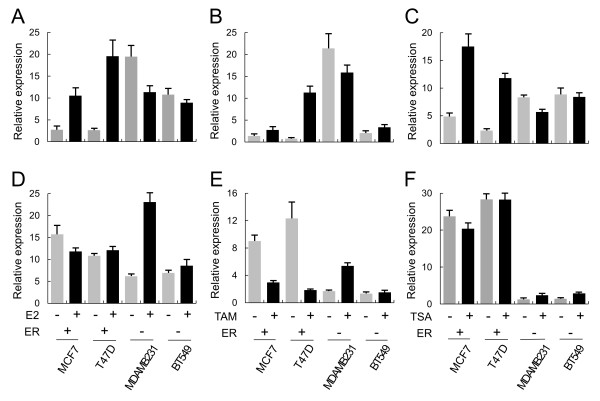
**Opposite effect of estradiol (E2), tamoxifen (TAM), and trichostatin A (TSA) on MTO1 vs. MRPL41 according to estrogen receptor (ER) status in breast cancer cell lines.** Cultured cells were treated with E2, TAM, and TSA, and MTO1 **(A–C)** and MRPL41 **(D–F)** expression levels were examined by real-time reverse transcription polymerase chain reaction. Gray and black bars represent before and after treatment with the indicated chemical. Each sample was examined in three independent reactions, and the average relative level is presented with the standard error.

As MTO1 and MRPL41 showed opposite expression patterns depending on ER status, we further examined the role of ER on their expression by monitoring the effect of an ER agonist and an antagonist. The agonist E2 increased MTO1 expression 3.9 and 7.4-fold in ER+ MCF7 and T47D cells, respectively, whereas it slightly decreased in ER- MDAMB231 and BT549 cells (Figure 4A). E2 increased MRPL41 gene expression 3.7 and 1.2-fold in ER- MDAMB231 and BT549 cells, whereas it induced a slight change with a 1.3-fold decrease and a 1.1-fold increase in ER+ MCF7 and T47D cells, respectively (Figure 4D).

The antagonist tamoxifen increased MTO1 expression 2 and 15-fold in ER+ cells, whereas it increased MRPL41 expression 3.2 and 1.1-fold in ER- cells (Figure 4B and E). However expression of the two genes in other ER cell type decreased in all cases, except MTO1 was increased slightly in BT549 cells.

The histone deacetylase inhibitor TSA was added to the cultured cells to induce histone acetylation and to examine the effect of chromatin structure on gene expression. Interestingly, TSA also induced the same pattern of expression change for the two genes in ER+ and ER- cells. MTO1 was increased 3.6 and 5-fold in ER+ cells, whereas MRPL41 was increased 1.9 and 2-fold in ER- cells (Figure 4C and F). Expression in the other cell types only decreased slightly.

Taken together, E2, tamoxifen, and TSA induced upregulation of MTO1 in ER+ cells while inducing upregulation of MRPL41 in ER- cells. The effect of the three chemicals in the other ER type cells was not remarkable, except for a slight downregulation.

### MTO1 and MRPL41 promoters are differentially regulated in ER+ and ER- cells

**Figure 5:**
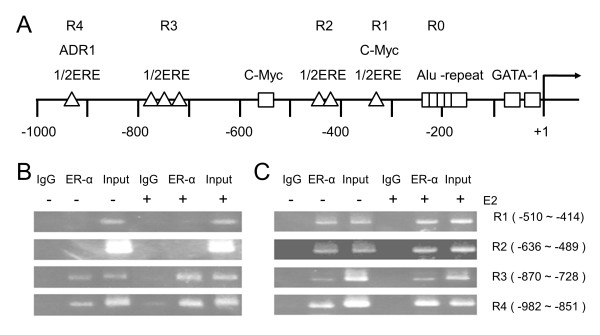
**Chromatin immunoprecipitation (ChIP) analysis of the MTO1 promoter against estrogen receptor (ER)-α.** ChIP assays were performed on the MTO1 promoter using anti-ER-α antibody followed by polymerase chain reaction (PCR) to amplify the half-ER-responsive element (1/2ERE) containing sub-regions. **(A)** Schematic diagram of the MTO1 promoter showing the four ER-responsive element (ERE) groups (R1-R4). The number of triangles denotes a tandem repeat of the 1/2ERE. Plausible binding sites for other transcriptional factors are also indicated. **(B and C)** Results of ChIP-PCR for the ER+ MCF7 cells **(B)** and the ER- MDAMB231 cell **(C)**. Cells were not treated (-) or treated (+) with estradiol.

We speculated that differential ER binding to the ER-responsive element (ERE) at the promoter could be a candidate molecular mechanism underlying the differential regulation of MTO1 and MRPL41 in ER+ and ER- cells. Thus, we first searched for EREs at the promoters of the two genes. As shown in Figure 5A, MTO1 had four groups of ERE-related sequences scattered over 1 kb upstream of the transcription start site with 1–3 repeats in each group. The perfect consensus sequence of ERE is GGTCAnnnTGACC, however, all EREs in MTO1 strikingly appeared as perfect or imperfect half-ERE (hERE) rather than a full ERE such as GGTCA, TGACC, GGCCA, and GGCAC. It has been known that the hERE is properly recognized by the ER [16].

**Figure 6:**
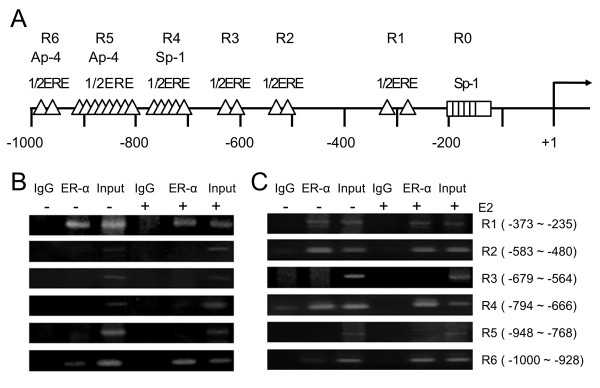
**Chromatin immunoprecipitation (ChIP) analysis of the MRPL41 promoter against estrogen receptor (ER)-α.** ChIP assays were performed on the MRPL41 promoter using anti-ER-α antibody followed by polymerase chain reaction (PCR) to amplify the half-ER-responsive element containing sub-regions. **(A)** Schematic diagram of the MRPL41 promoter showing the six ER-responsive element (ERE) groups (R1–R6). The number of triangles denotes tandem repeat of the 1/2ERE. Plausible binding sites for other transcriptional factors are also indicated. **(B and C)** Results of ChIP-PCR for ER+ MCF7 cells **(B)** and ER- MDAMB231 cells **(C)**. Cells were not treated (-) or treated (+) with estradiol.

ChIP analysis of the MTO1 promoter determined that among the R1–R4 hEREs, only R3 and R4 were bound to ER-α in ER+ MCF7 cells (Figure 5B). However, R1 and R2 were also bound to ER-α as well as R3 and R4 in ER- MDAMB231 cells (Figure 5C). These differences in ER binding profiles may partly explain the opposite expression pattern between ER+ and ER- cells. There did not appear to be any considerable effect of E2 on the ER binding of both cell types. MRPL41 had six groups (R1–R6 in Figure 6A) of hEREs scattered within 1 kb of the promoter region with 2–8 repeats. Their sequences appeared as GGGCA, TGACC, or GGTGG. ChIP analysis of the PRPL41 promoter that had driven higher expression in ER- cells generally showed less ER binding compared to that of MTO1. Only R1 showed a remarkable level of binding in the ER+ MCF7 cells (Figure 6B), whereas R2 and R4 additionally bound in ER- MDAMB231 cells (Figure 6C). When E2 was added to the culture, new binding to R6 emerged in both cell types.

**Figure 7:**
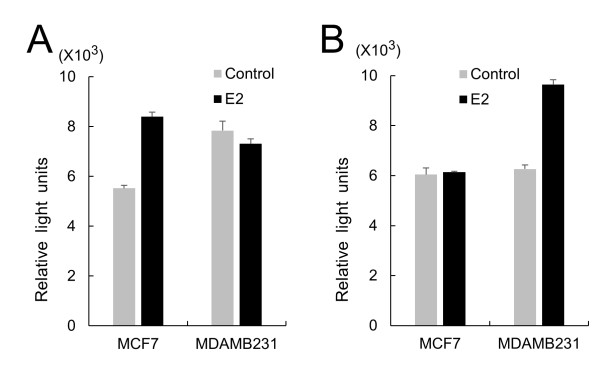
**Opposite activation of MTO1 and MRPL41 promoter by E2 in ER+ and ER- breast cancer cells.** Upstream regions of MTO1 (from -994 to +18) **(A)** and MRPL41 (from -1,030 to +1) **(B)** were placed upstream of the luciferase gene, and luciferase activities were determined from transiently transfected ER+ (MCF7) and ER- (MDAMB231) cells. Cells were not treated (gray bar) or treated (black bar) with estradiol (E2). Each experiment was performed at least three times and the data are presented as the average and standard error after normalization with activity from vectors containing R0 region in each gene.

To further analyze the effect of hEREs on the differential regulation of MTO1 and MRPL41 in ER+ and ER- cells, activity of the promoter containing the hEREs was measured using a luciferase reporter gene in MCF7 and MDAMB231 cells cultured with or without E2. When the cells were treated with E2, the MTO1 promoter containing the R1 ~ R4 regions significantly increased the reporter activity in the MCF7 cell, meanwhile the MRPL41 promoter containing the R1 ~ R6 regions significantly increased the reporter activity in the MDAMB231 cell (Figure 7). These results support the fact that the two genes are upregulated by E2 in the opposite ER cell types as indicated in Figure 4.

## Discussion

Promoter methylation and histone modification of cancer-related genes have played essential roles during carcinogenesis [17-20]. Recent data suggest that epigenetic status of breast cancer may undergo changes mediated by the action of estrogens and could also be affected by ER status [21,22]. The present results indicate that the two mitochondrial genes, MTO1 and MRPL41, were differentially regulated in breast cancer such that they showed the opposite response to E2, tamoxifen, and TSA. Our findings suggest that the opposite pattern of promoter methylation and differential binding of the ER to the promoter in both genes are explanations for this phenomenon.

In previous studies, a group of genes was regulated by the ER, and the majority of them were upregulated in response to estrogens whereas only a few genes including NFκB and CXCR7 were downregulated in response to estrogens [23,24]. However, no nuclear-encoded mitochondrial genes are known in terms of estrogen response, and this is the first study that has reported epigenetic regulation of mitochondrial genes in breast cancer according to ER status. Surprisingly, MRPL41 was upregulated by E2 in the MDAMB231 cell that was ER negative. It has been known that alternative signaling pathways were activated in ER- cancer cells. For example, estrogen is able to trigger signaling through receptors other than ER such as GPR30, upregulating target genes like c-fos [25]. Related with this fact, it is speculated that MRPL41 could be upregulated by alternative receptors other than ER.

The ER antagonist tamoxifen also stimulated expression of MTO1 in ER+ cells similar to E2 and TSA. This estrogen-like stimulatory effect of tamoxifen has also been found in several other genes such as Heparinase and PTPRO [26,27], providing an explanation for altered tamoxifen activity from an antagonist to an agonist. This result suggests that tamoxifen acts as an MTO1 agonist in ER+ cells, but as an MRPL41 antagonist in ER- cells. Detailed understanding of the mechanism through which estrogen and tamoxifen affect MTO1 and MRPL41 transcription is expected to provide new insights into breast cancer progression and suggest new strategies for delaying or reversing this process.

It is thought that upregulation of MTO1 by TSA in ER+ cells may be linked to promoter demethylation. Previous studies support this hypothesis, where histone hypermethylation induces demethylation of promoters and thereby upregulates gene expression [28,29]. We also found that TSA induced demethylation in the ER+ cells which had shown hypermethylation and downregulation of MTO1 (Additional file 5: Figure S3). Therefore, histone acetyl transferase (or deacetylase) and CpG methyltransferase may act together to regulate gene expression on the MTO1 promoter in the ER+ cells.

In this study, the hERE sites scattered at the MTO1 and MRPL41 promoters appropriately bound the ER. The two genes responded differently according to ER status in both breast tissues and cultured cells. However, they did not show any significant changes in response to E2, suggesting that other elements are required for the complete regulation of ER binding. In fact, similar to other E2 responsive genes expressed in human breast cancer cells such as cathepsin D, c-fos, and c-myc [30-32], the MRPL41 upstream promoter region has two Sp1/Sp3 binding site near hERE sites and five tandem repeats just downstream of the R1 region. Two c-myc sites, instead of Sp1 sites, are nested in hERE sites in MTO1. Previous studies suggested that E2 stimulation results in the recruitment of the transcription factors ERα, Sp1, and Sp3 to the promoter [33-35]. However, further examination should be carried out to elucidate the precise mechanism of how each hERE acts to stimulate the two genes because our results show that the hEREs used a different platform of transcriptional factor recognition elements, and were differentially regulated according to ER status.

It should be mentioned that the upregulated pattern of the two genes in breast cancer shown by DDD was not repeated in our patient tissues. It is speculated that the EST hits registered at the database were too small to show statistical significance or that the ESTs were largely extracted from cancer tissues. In addition, even though there appeared to be a significant difference, both normal and cancer tissues generally showed lower methylation levels when examined by methylation-specific PCR. One explanation could be due to a mix-up of normal cells with cancer cells during surgery. In fact the cancer cell lines showed much higher methylation level than the cancer tissues. Otherwise, other CpGs with higher methylation might be missed because methylation-specific PCR compared only four CpG sites. A detailed understanding of the molecular events occurring along opposite pathways will provide more comprehensive insight into the biology of estrogen-driven breast tumorigenesis in the case of mitochondrial genes and may have important implications for recommendations on treatment and risk-reduction strategies.

## Conclusions

In conclusion, nuclear-encoded mitochondrial MTO1 and MRPL41 showed an opposite expression pattern according to estrogen receptor (ER) status. MTO1 was upregulated in ER- cancer types, meanwhile MRPL41 was upregulated in ER+ cancer types, showing an inverse correlation between expression and promoter methylation. Furthermore, modifiers of ER (E2 and tamoxifen) and histone deacetylase (TSA) also induced the two genes in an opposite mode in the ER+ and ER- cell types. Differential binding and influencing of ER to the promoter is involved in the differential regulation. Taken together, identifying the link between epigenetic regulation and MTO1 and MTRL41 expression may represent novel breast cancer markers that are regulated in opposite ways by ER modulators.

## Abbreviations

CpG: Cytosine guanine dinucleotide; DDD: Digital differential display; ER: Estrogen receptor; ERE: Estrogen-responsive element; MSP: Methylation specific PCR; RT-PCR: Reverse transcription-polymerase chain reaction.

## Competing interests

The authors declare that they have no competing interests.

## Authors’ contributions

Conceived and designed the experiments: HSK SJK. Performed the experiments: TWK BK JHK SK SJK. Analyzed the data: GJ SBP HSK SJK. Wrote the paper: SJK. All authors read and approved the final manuscript.

## Pre-publication history

The pre-publication history for this paper can be accessed here:

http://www.biomedcentral.com/1471-2407/13/502/prepub

## Supplementary Material

Additional file 1: Table S1 Sequences of primers employed in this study. Click here for file

Additional file 2: Table S2 Top 10 genes with highest enrichment in breast identified by EST profile. Click here for file

Additional file 3: Figure S1 Methylation and expression of MTO1 and MRPL41 in breast cancer tissues according to the ER status. Methylation and expression of MTO1 (A and B) and MRPL41 (C and D) were examined by real-time MSP and RT-PCR, respectively in ER(+) and ER(-) breast cancer tissues. N in parenthesis denotes the number of examined tissues. Each sample was examined in duplicate and the average was applied to the plot. Click here for file

Additional file 4: Figure S2 Methylation status of CpG islands at the promoter of MTO1 and MRPL41 in breast cancer cell lines. Schematic diagram of the promoter is presented with the CpG region of which methylation status was determined by MSP and bisulfite sequencing. CpG sites were denoted by vertical lines in red at the top. Methylation status determined by direct sequencing was denoted by circles. Sequencing diagrams corresponding to different methylation levels are presented at the bottom. (A) MTO1. (B) MRPL41. (C) A1BG and ETAA1. Click here for file

Additional file 5: Figure S3 Change of methylation level of MTO1 and MRPL41 according to the ER status after TSA treatment. Methylation of MTO1 (A) and MRPL41 (B) were examined by real-time MSP in ER(+) and ER(-) breast cancer cell lines after treatment of TSA. Each sample was examined in three independent reactions, and the average level was presented with the standard error. Click here for file
