# Supplementary material for: Nuclear-encoded mitochondrial MTO1 and MRPL41 are regulated in an opposite epigenetic mode based on estrogen receptor status in breast cancer
Source: BMC Cancer. 2013 Oct 27;13:502. doi: 10.1186/1471-2407-13-502 (PMC4015551; doi:10.1186/1471-2407-13-502)
Supplement: Additional file 1: Table S1 — Sequences of primers employed in this study. [file 1471-2407-13-502-S1.doc]

| **Table S1. Sequences of primers employed in this study** | | | | | |
| --- | --- | --- | --- | --- | --- |
| **Genes** | | **Forward primer (5’-3’)** | | **Reverse primer (5’-3’)** | |
| **Real-time RT-PCR** | | | |  | |
| MTO1 | | TCTATAAACAGAACATGCAGAAAGAAA | | GTGCTTCCATCCACCAAAA | |
| MRPL41 | | AGCTCGTGTGGGGTCTCC | | AGGTGAGGAAGCCGATGC | |
|  | |  | |  | |
| **Real-time MSP** | | | |  | |
| MTO1 M | | AATTAGGAAAGAAAATATATAATCGG | | TATCACGAAATAAAACGTCTACGTC | |
| MTO1 U | | GAATTAGGAAAGAAAATATATAATTGG | | TATCACAAAATAAAACATCTACATC | |
| MRPL41 M | | GTAGGAGGGTTTAGTGCGTAAGTC | | AACCGAACAATAATATAAAAAAACTCG | |
| MRPL41 U | | GTAGGAGGGTTTAGTGTGTAAGTTG | | AACCAAACAATAATATAAAAAAACTCAAA | |
|  | |  | |  | |
| **PCR after ChIP** | | | |  | |
| MTO1 R1 | | GTAGGAGCTCCCAACCCAGA | | CCGTATTAGCCAGGATGTTC | |
| MTO1 R2 | | GGTGACGGTGTATCAGATTG | | TCTGGGTTGGGAGCTCCTAC | |
| MTO1 R3 | | GCTGGGATTACAGGTGCATG | | GGCCGAAGCAGGTGGATCAC | |
| MTO1 R4 | | GAGACGGAGTCTTGCTCTGT | | CATGCACCTGTAATCCCAGC | |
| MRPL41 R1 | | TCTCGAGTCCAGGGCCCGCG | | CCGTATTAGCCAGGATGTTC | |
| MRPL41 R2 | | GGTGACGGTGTATCAGATTG | | CCGGGAGGGCCCGGGCTGTC | |
| MRPL41 R3 | | GATTGTGAGGGTGCGGCTGG | | CAATAATCTTGGACCCAGCA | |
| MRPL41 R4 | | TGGATCTGGGGTGTGGGGGT | | GATCCGGATTGTGAGGGTGC | |
| MRPL41 R5 | | CTGGGGTGTGGGGGTGCCGC | | CCAGCGGTACCCCCACACCC | |
| MRPL41 R6 | | CGGCGAGTGGGGGTACCGCT | | GCGGCACCCCCACACCCCAG | |
| **PCR for luciferase** | | |  | |  |
| MTO1 R0 | ACGCGTGAGGCTAAGGCAGG | | AAGCTTACAATCTGCAGGGC | |  |
| MTO1 R4 | ACGCGTGAGTCTTGCTCTGT | | AAGCTTACAATCTGCAGGGC | |  |
| MRPL41 R0 | ACGCGTGAGAGCGATCACTC | | AAGCTTCGTCCAATAGCGGC | |  |
| MRPL41 R6 | ACGCGTGTGCAGCTGGGGAG | | AAGCTTTAGCGGCCCCAATC | |  |
| **Bisulfite sequencing** | | |  | |  |
| MTO1 | GAGGGGGAGTTTGTAGTGAG | | CCTCCACCAATAACTATCAC | |  |
| MRPL41 | GGTTTTAGGGGGATTGGGTT | | CCCCAATCTAATAAAAAATA | |  |
|  |  | |  | |  |
|  |  | |  | |  |
