# Supplementary material for: Nuclear-encoded mitochondrial MTO1 and MRPL41 are regulated in an opposite epigenetic mode based on estrogen receptor status in breast cancer
Source: BMC Cancer. 2013 Oct 27;13:502. doi: 10.1186/1471-2407-13-502 (PMC4015551; doi:10.1186/1471-2407-13-502)
Supplement: Additional file 2: Table S2 — Top 10 genes with highest enrichment in breast identified by EST profile. [file 1471-2407-13-502-S2.doc]

| **Table S2. Top 10 genes with highest enrichment in breast identified by EST profile** | | | | | |
| --- | --- | --- | --- | --- | --- |
| Unigene  ID No. | EST profile | | Symbol | Enrichment in cancer^a^ | Presence of CpG islands^b^ |
|  | Breast cancer | Mammary gland |  |  |  |
| Hs.646899 | 1 | 107 | Ellis van Creveld syndrome (EVC) | N | + |
| Hs.654465 | 0 | 74 | Glutamate-cysteine ligase, catalytic subunit (GCLC) | N | + |
| Hs.529161 | 0 | 53 | Alpha-1-B glycoprotein (A1BG) | N | + |
| Hs.353022 | 2 | 116 | Ewing tumor-associated antigen 1 (ETAA1) | N | + |
| Hs.44017 | 113 | 121 | Mitochondrial ribosomal proteinL41 (MRPL41) | C | + |
| Hs.347614 | 96 | 96 | Mitochondrial translation optimization 1 homolog (MTO1) | C | + |
| Hs.560938 | 81 | 98 | Melanophilin (MLPH) | C | + |
| Hs.439060 | 1 | 44 | Claudin1 (CLDN1) | N | + |
| Hs.535731 | 1 | 30 | Protein Phosphatase1, regulatory(inhibitor)subunit2 (PPP1R2) | N | + |
| Hs.355581 | 1 | 10 | Zinc finger and BTB domain containing43 (ZBTB43) | N | + |
| a. Frequency of ESTs is high in cancer (C), or in normal breast tissue (N). | | | | | |
| b. Presence (+) or absence (-) of CpG islands in the promoter | | | | | |
