# Supplementary material for: Nuclear-encoded mitochondrial MTO1 and MRPL41 are regulated in an opposite epigenetic mode based on estrogen receptor status in breast cancer
Source: BMC Cancer. 2013 Oct 27;13:502. doi: 10.1186/1471-2407-13-502 (PMC4015551; doi:10.1186/1471-2407-13-502)

**Fig. S1. Methylation and expression of MTO1 and MRPL41 in breast cancer tissues according to the ER status.** Methylation and expression of MTO1 (A and B) and MRPL41 (C and D) were examined by real-time MSP and RT-PCR, respectively in ER(+) and ER(-) breast cancer tissues. N in parenthesis denotes the number of examined tissues. Each sample was examined in duplicate and the average was applied to the plot.


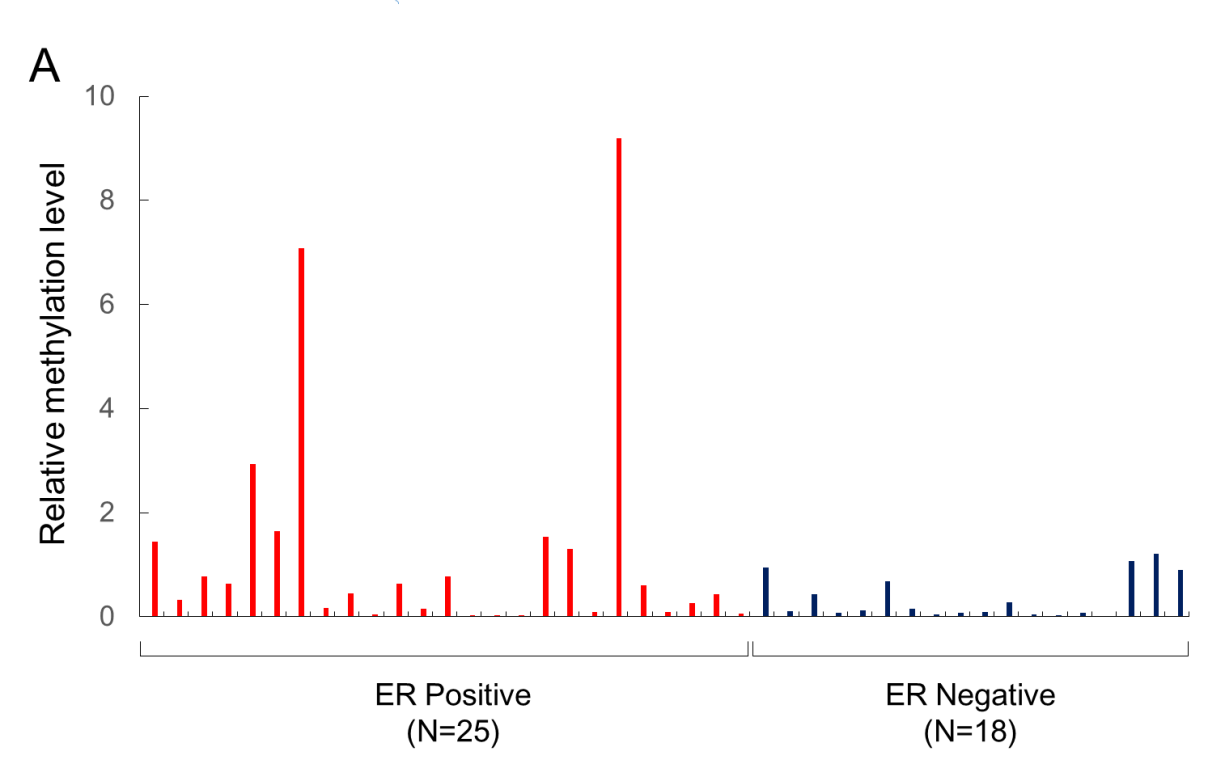


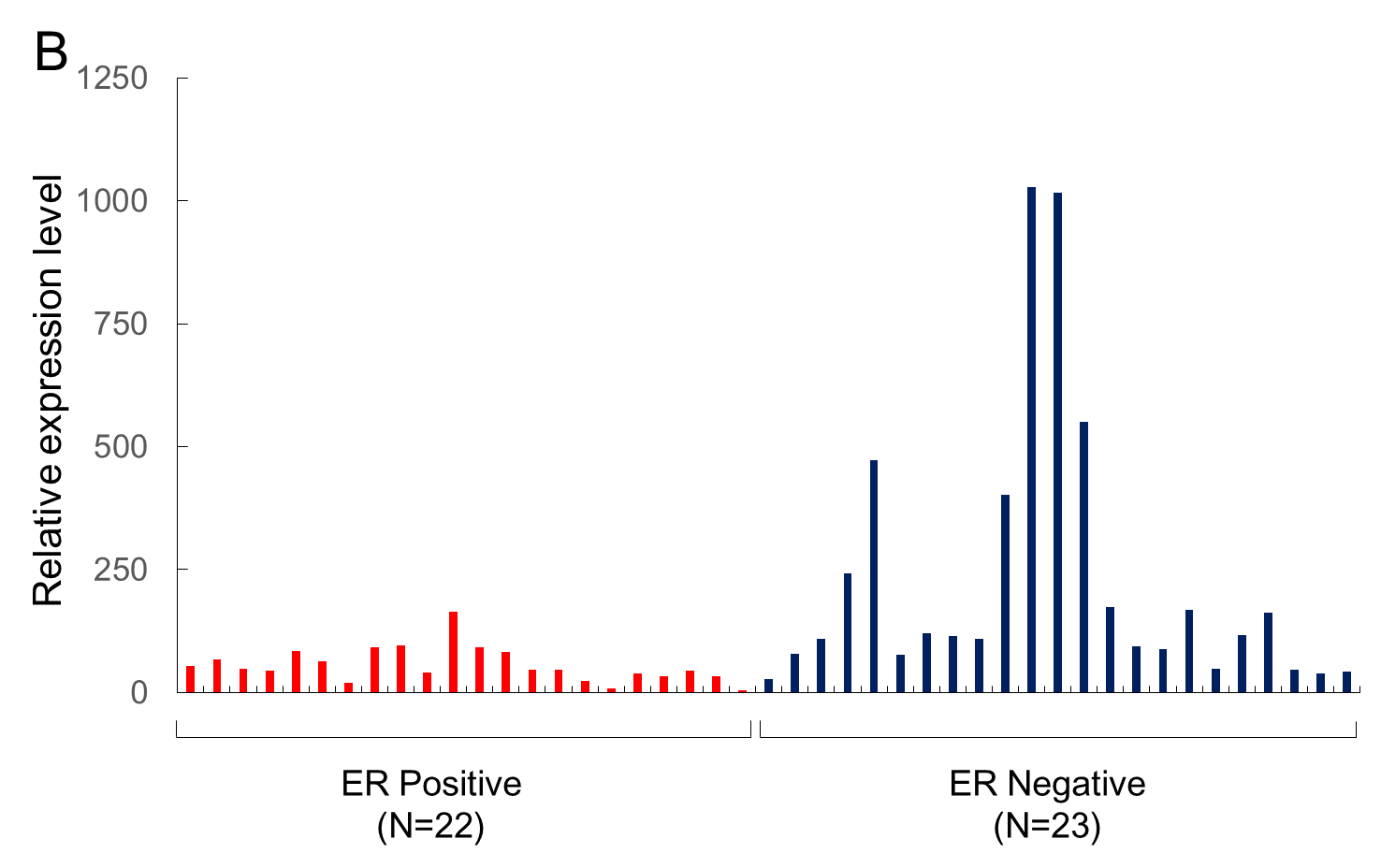


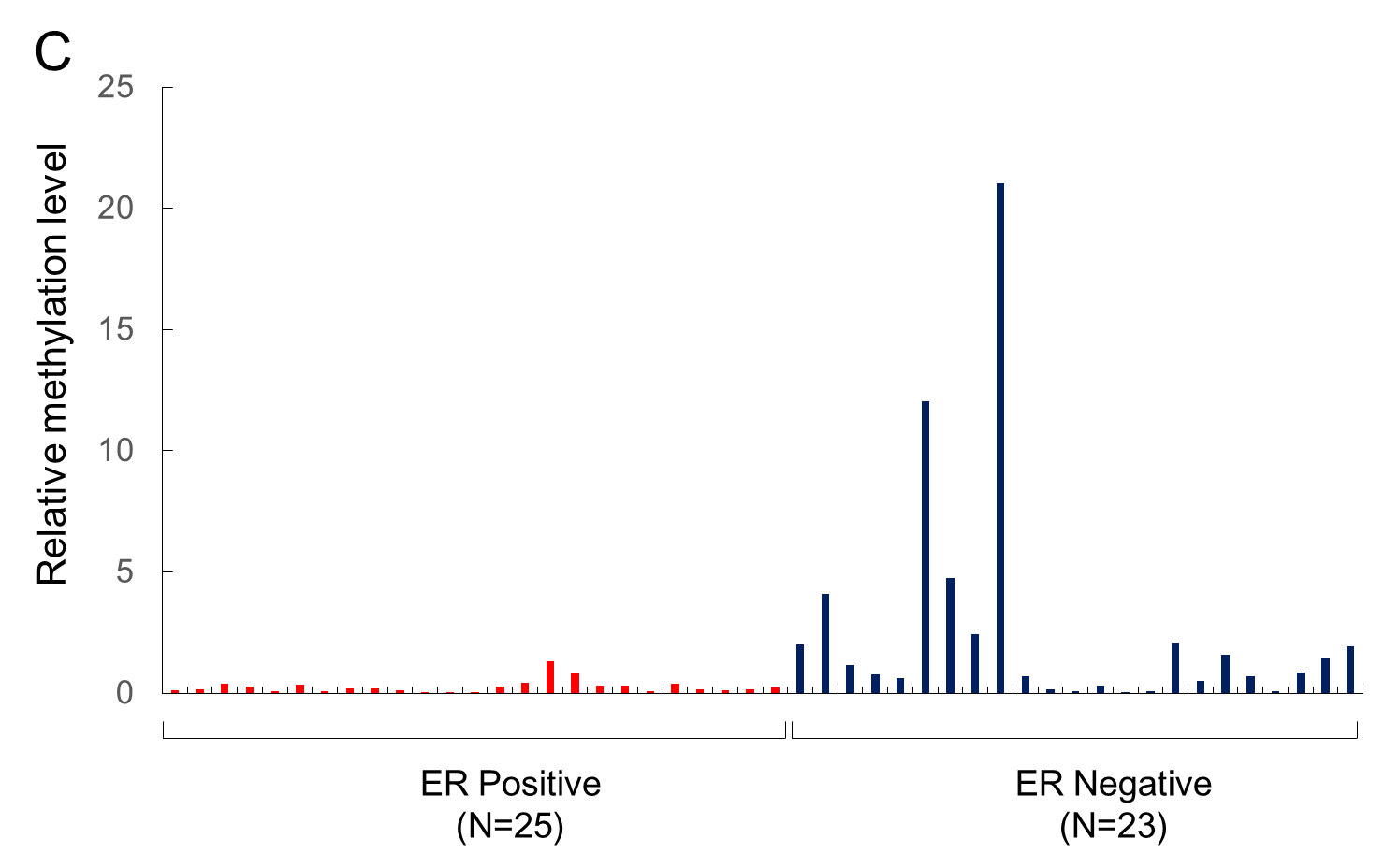


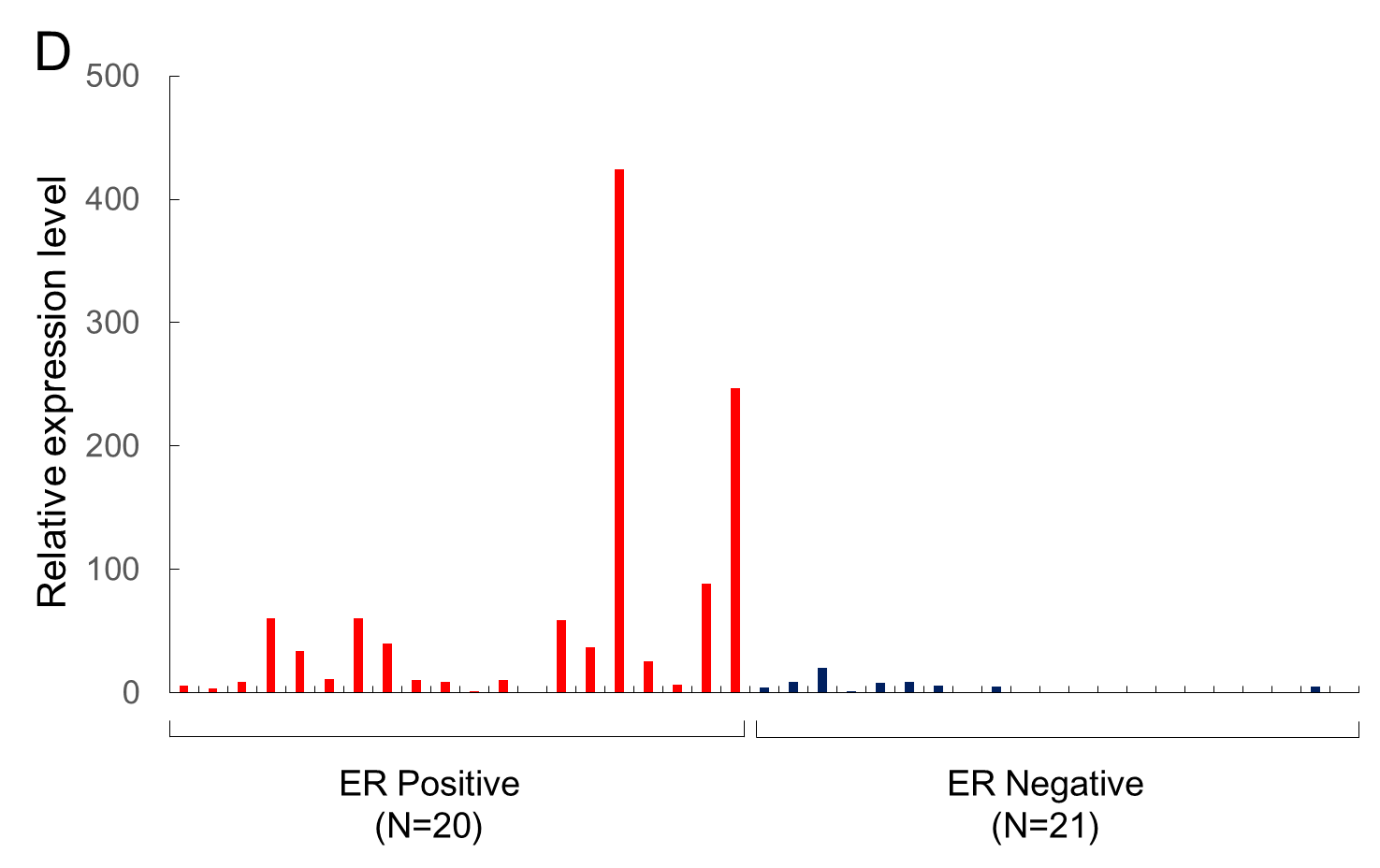

Supplement: Additional file 3: Figure S1 — Methylation and expression of MTO1 and MRPL41 in breast cancer tissues according to the ER status. Methylation and expression of MTO1 (A and B) and MRPL41 (C and D) were examined by real-time MSP and RT-PCR, respectively in ER(+) and ER(-) breast cancer tissues. N in parenthesis denotes the number of examined tissues. Each sample was examined in duplicate and the average was applied to the plot. [file 1471-2407-13-502-S3.doc]
