# Supplementary material for: Nuclear-encoded mitochondrial MTO1 and MRPL41 are regulated in an opposite epigenetic mode based on estrogen receptor status in breast cancer
Source: BMC Cancer. 2013 Oct 27;13:502. doi: 10.1186/1471-2407-13-502 (PMC4015551; doi:10.1186/1471-2407-13-502)

**Fig. S2. Methylation status of CpG islands at the promoter of MTO1 and MRPL41 in breast cancer cell lines.** Schematic diagram of the promoter is presented with the CpG region of which methylation status was determined by MSP and bisulfite sequencing. CpG sites were denoted by vertical lines in red at the top. Methylation status determined by direct sequencing was denoted by circles. Sequencing diagrams corresponding to different methylation levels are presented at the bottom. (A) MTO1. (B) MRPL41. (C) A1BG and ETAA1.


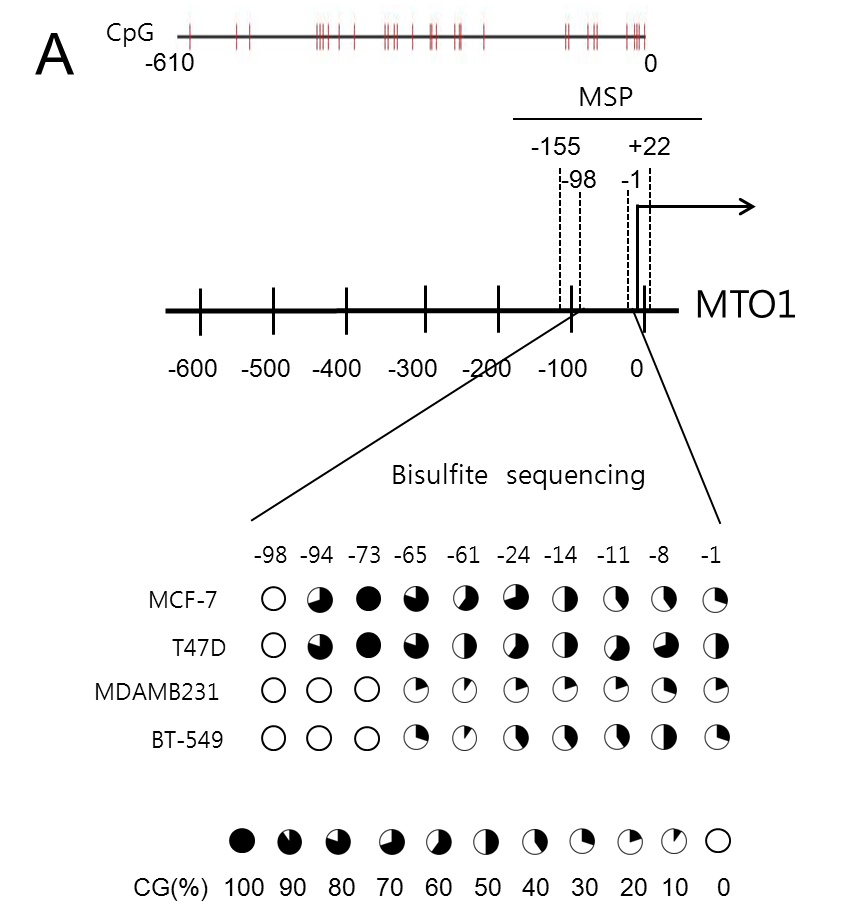


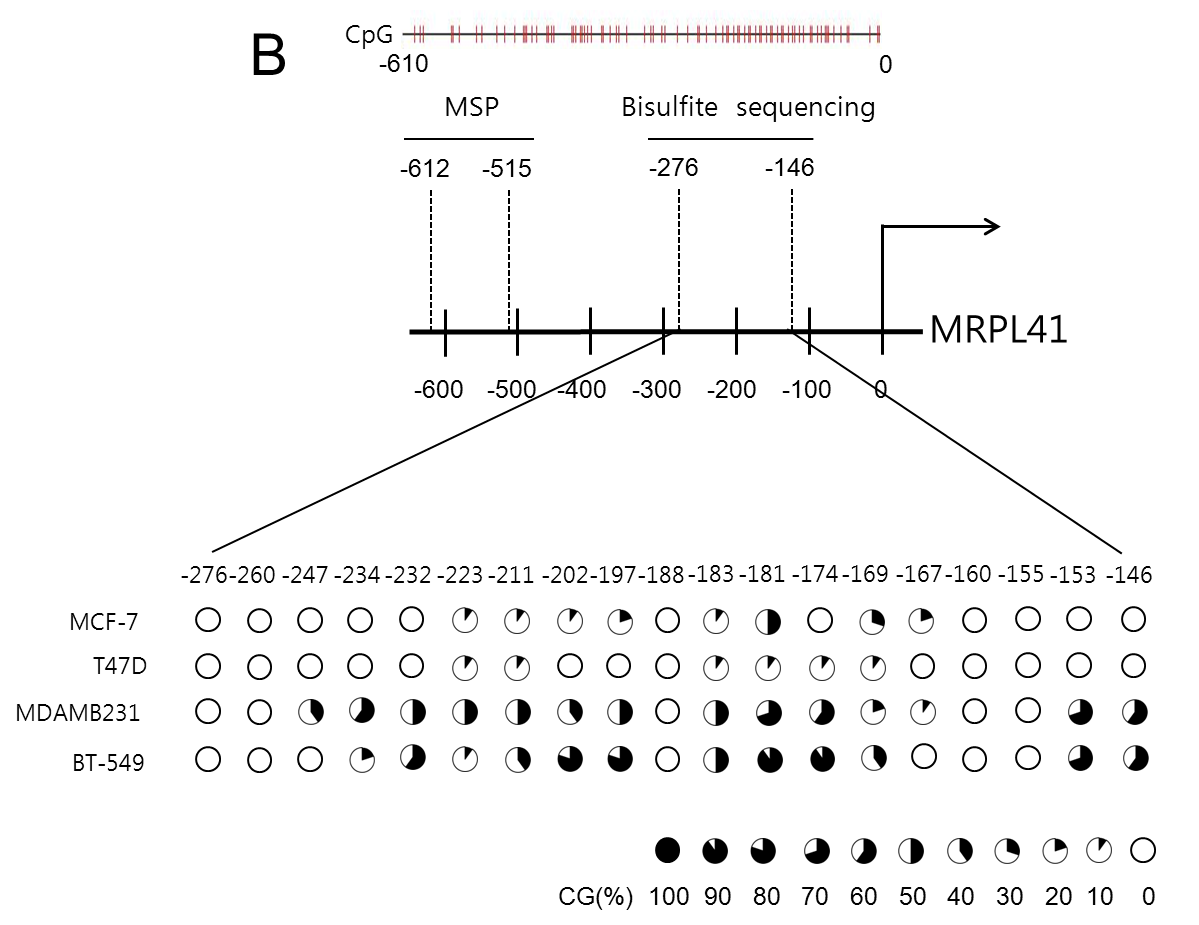


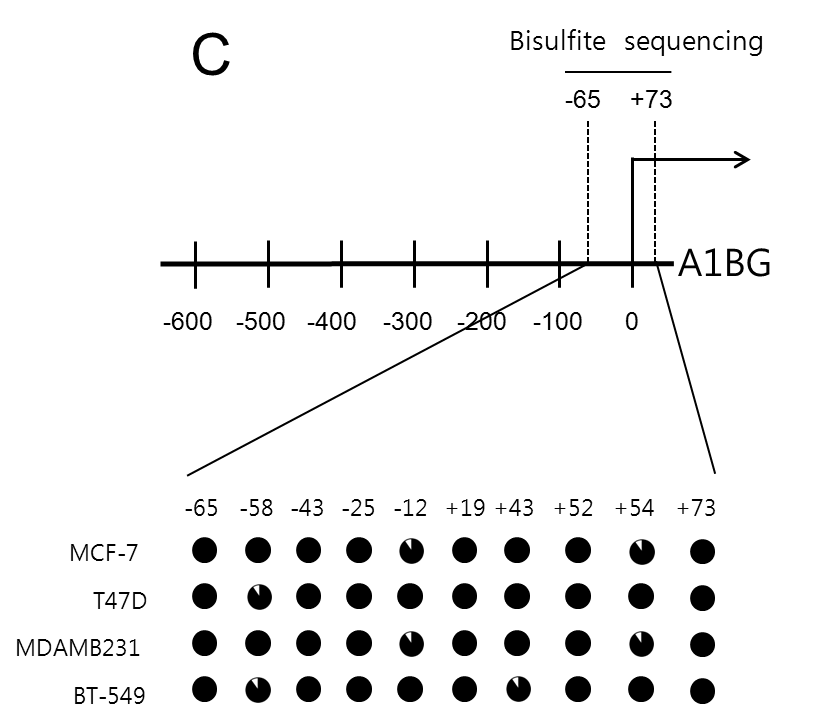


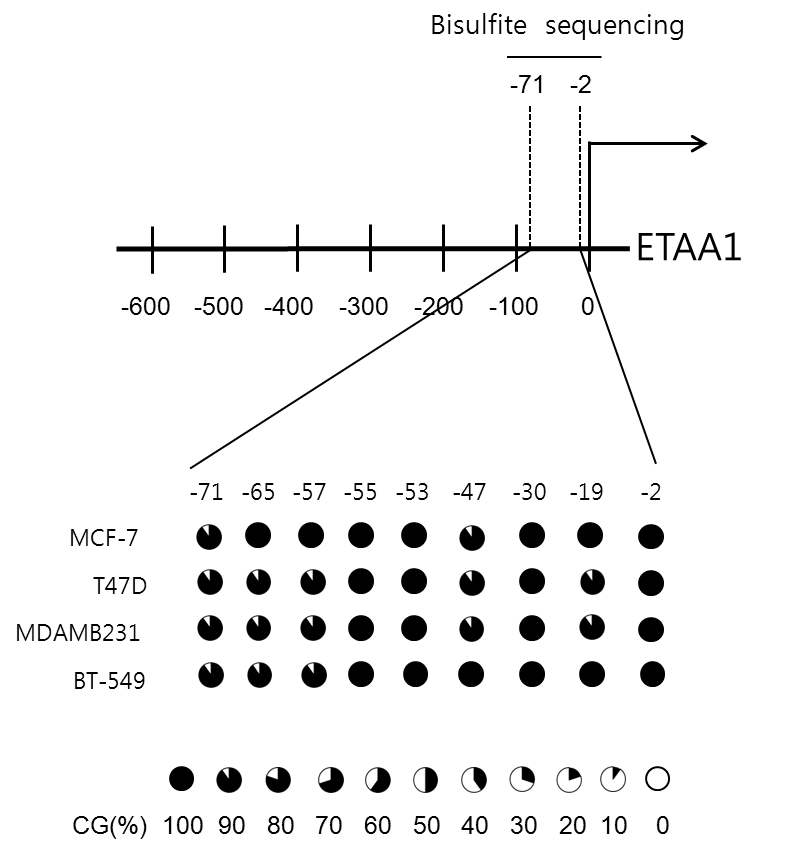

Supplement: Additional file 4: Figure S2 — Methylation status of CpG islands at the promoter of MTO1 and MRPL41 in breast cancer cell lines. Schematic diagram of the promoter is presented with the CpG region of which methylation status was determined by MSP and bisulfite sequencing. CpG sites were denoted by vertical lines in red at the top. Methylation status determined by direct sequencing was denoted by circles. Sequencing diagrams corresponding to different methylation levels are presented at the bottom. (A) MTO1. (B) MRPL41. (C) A1BG and ETAA1. [file 1471-2407-13-502-S4.doc]
