# Supplementary material for: Nuclear-encoded mitochondrial MTO1 and MRPL41 are regulated in an opposite epigenetic mode based on estrogen receptor status in breast cancer
Source: BMC Cancer. 2013 Oct 27;13:502. doi: 10.1186/1471-2407-13-502 (PMC4015551; doi:10.1186/1471-2407-13-502)

**Fig. S3. Change of methylation level of MTO1 and MRPL41 according to the ER status after TSA treatment.** Methylation of MTO1 (A) and MRPL41 (B) were examined by real-time MSP in ER(+) and ER(-) breast cancer cell lines after treatment of TSA. Each sample was examined in three independent reactions, and the average level was presented with the standard error.


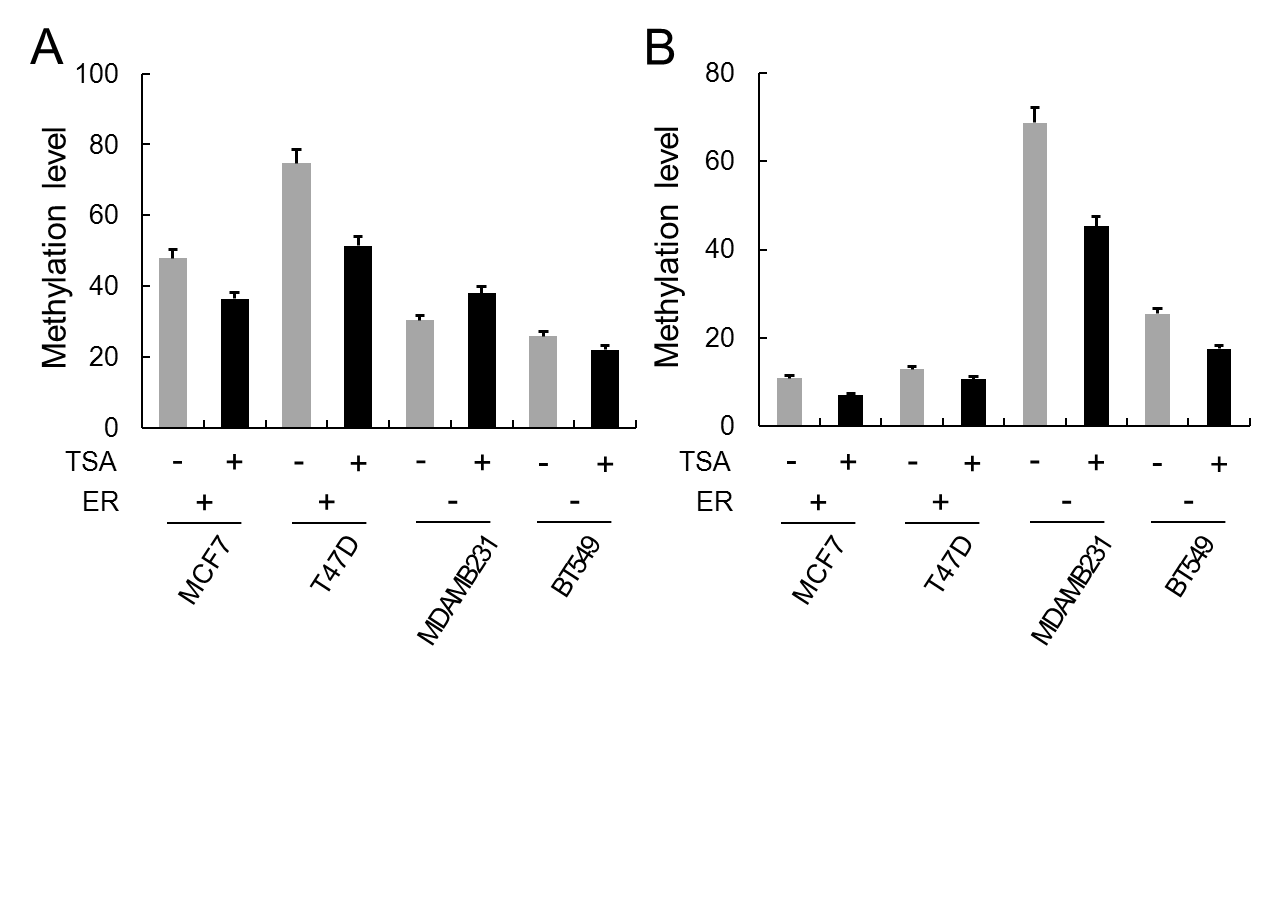

Supplement: Additional file 5: Figure S3 — Change of methylation level of MTO1 and MRPL41 according to the ER status after TSA treatment. Methylation of MTO1 (A) and MRPL41 (B) were examined by real-time MSP in ER(+) and ER(-) breast cancer cell lines after treatment of TSA. Each sample was examined in three independent reactions, and the average level was presented with the standard error. [file 1471-2407-13-502-S5.doc]
